# Neurobiological Pathways Underlying Opioid Addiction and Overdose: Mechanisms, Therapeutic Targets, and Insights from Zebrafish Model

**DOI:** 10.3390/ijms27209339

**Published:** 2026-10-21

**Authors:** Jovana Duric, Frederick E. Williams, Isaac T. Schiefer

**Affiliations:** 1 Department of Pharmacology and Experimental Therapeutics, College of Pharmacy and Pharmaceutical Sciences, University of Toledo, Toledo, OH 43606, USA; jovana.duric@rockets.utoledo.edu (J.D.);; 2 Center for Drug Design and Development, College of Pharmacy and Pharmaceutical Sciences, University of Toledo, Toledo, OH 43606, USA; 3 Department of Medicinal and Biological Chemistry, College of Pharmacy and Pharmaceutical Sciences, University of Toledo, Toledo, OH 43606, USA

**Keywords:** opioid addiction, overdose, fentanyl, μ-opioid receptor, biased agonism, neurobiology, zebrafish, CRISPR-Cas9, respiratory depression

## Abstract

The worldwide opioid epidemic continues to claim thousands of lives each year and remains one of the most urgent global public health challenges, driven by the high abuse potential and overdose risk of potent, fully synthetic μ-opioid receptor (MOR) agonists such as fentanyl. While fentanyl is highly effective in pain management, it also carries severe risks including respiratory depression, addiction, and fatal overdose. Understanding the neurobiological pathways underlying these risks is essential for developing safer analgesics and effective interventions. The MOR, a G-protein-coupled receptor (GPCR) that modulates neuronal excitability and synaptic transmission in important brain circuits like the ventral tegmental area (VTA), nucleus accumbens (NAc), and prefrontal cortex (PFC), is the primary receptor through which opioids exert their therapeutic effects. Dopaminergic dysregulation, transcriptional remodeling, and altered synaptic plasticity are neuroadaptations brought on by long-term opioid exposure that contribute to dependence and relapse. Life-threatening opioid toxicity is primarily driven by MOR-mediated suppression of respiratory networks, with risk shaped by dose, pharmacokinetics, receptor efficacy, and circuit sensitivity. Zebrafish (*Danio rerio*) provide a powerful complementary system for dissecting opioid-related neurocircuitry and screening for potential therapeutics. Zebrafish share conserved opioid-signaling components with mammals, and larval transparency facilitates in vivo imaging of neuronal activity at cellular resolution. Behavioral paradigms capture reward-related and drug-seeking phenotypes, while CRISPR-based approaches offer a means to investigate receptor-specific functions in validated models. In addition, zebrafish larvae provide an efficient platform for high-throughput screening of compounds that modulate opioid signaling and respiratory-related phenotypes. Together, insights from human, rodent, and zebrafish studies provide a more integrated understanding of opioid overdose and addiction neurobiology. This review incorporates current knowledge on the molecular and circuit-level mechanisms driving these processes and discusses how zebrafish models accelerate the discovery of safer analgesics and novel treatments for opioid use disorder and overdose.

## 1. Introduction

The opioid crisis remains one of the most pressing public health emergencies worldwide, claiming hundreds of lives and imposing an immense social and economic burden. The opioid crisis was initially fueled by overprescription of opioid analgesics in the late 1990s and evolved through consecutive waves [1]. It began with the misuse of prescription opioids, followed by heroin, and is now dominated by synthetic opioids and their analogs. Opioid-related overdose remains a major cause of mortality in the United States [1]. This dangerous direction shows the urgent need for a comprehensive understanding of neurobiological mechanisms underlying opioid addiction and overdose.

The term opioid encompasses natural, semi-synthetic, and fully synthetic compounds that act at opioid receptors, including the three classical receptors, μ (mu), δ (delta), and κ (kappa), in the central and peripheral nervous systems [2,3]. In contrast, opiates refer to naturally occurring alkaloids derived from the opium poppy (*Papaver somniferum*), such as morphine and codeine. Semi-synthetic opioids, including heroin, oxycodone, and hydrocodone, are chemically modified natural compound derivatives, while fully synthetic opioids are entirely manmade. The term opioid is now widely used in both clinical and research contexts, encompassing all agents, no matter the origin, that exert their effects through opioid receptor signaling. Fentanyl, methadone, and tramadol are some of the most popular synthetic opioids acting on the same receptor system, the μ-opioid receptor (MOR), which belongs to the membrane-bound G-protein-coupled receptor (GPCR) family found in the central nervous system and partially in peripheral tissues [3]. MOR activation mediates analgesia, respiratory depression, and reinforcing effects and contributes to physical dependence after repeated exposure [2,3]. Chronic exposure to opioids causes adaptive changes in dopaminergic, glutamatergic, and GABAergic signaling in key brain regions: the ventral tegmental area (VTA), nucleus accumbens (NAc), and prefrontal cortex (PFC). These neuroadaptations alter synaptic plasticity, gene expression, and receptor signaling, contributing to the transition from controlled drug use to uncontrollable addiction. Over time, opioids have evolved into a broad range of therapeutics used in pain management, especially in cases of cancer, surgery, trauma, and palliative care. The clinical use of opioids is limited by their potential for misuse, addiction, tolerance, and fatal outcomes. Developmental stage and social context should also be considered when interpreting opioid responses and patterns of use across age groups.

Moreover, opioid overdose represents the most severe and life-threatening consequence of opioid misuse. Fatal overdose results from MOR-mediated inhibition of respiratory centers in the brainstem, leading to respiratory depression [4,5]. Naloxone is an opioid-receptor antagonist used to reverse opioid-induced respiratory depression. Because opioid effects can outlast naloxone, repeated dosing may be needed when respiratory depression persists or recurs [4]. The development of novel therapeutics that maintain analgesic efficacy while minimizing respiratory and addictive side effects is necessary.

Under the U.S. Controlled Substances Act, drugs are assigned to five schedules according to their accepted medical use and abuse or dependence potential. Schedule I substances, such as heroin and LSD, have no currently accepted medical use in the United States and a high potential for abuse. Schedule II substances, including methadone, fentanyl, oxycodone, and morphine, have accepted medical uses, but a high abuse potential. Schedule III substances, such as ketamine and anabolic steroids, have a lower abuse potential than Schedule I or II substances. Schedule IV substances, including alprazolam, clonazepam, and diazepam, have a lower abuse potential than Schedule III substances. Schedule V substances have a lower abuse potential than Schedule IV substances and include certain low-codeine cough preparations and ezogabine [6].

Traditional mammalian and rodent models have been instrumental in uncovering the cellular and circuit-level underpinnings of opioid action; however, they are limited by cost, scalability and accessibility for high-throughput pharmacological studies. Therefore, zebrafish (*Danio rerio*) have emerged as a powerful model to investigate molecular and behavioral effects of opioid exposure [7]. Approximately 70% of human protein-coding genes have at least one zebrafish orthologue. However, 84% of genes associated with human diseases have zebrafish-equivalent genes [8]. Zebrafish offer a unique combination of genetic tractability, conserved neurochemical pathways, and high-throughput behavioral and imaging capabilities that enable rapid exploration of addiction-related phenotypes and therapeutic screening, making them a valuable tool for pharmacological research and drug screening [9].

## 2. Opioid Receptors and Signaling

### 2.1. MOR Structure, Signaling and Key Domains

**Figure 1:**
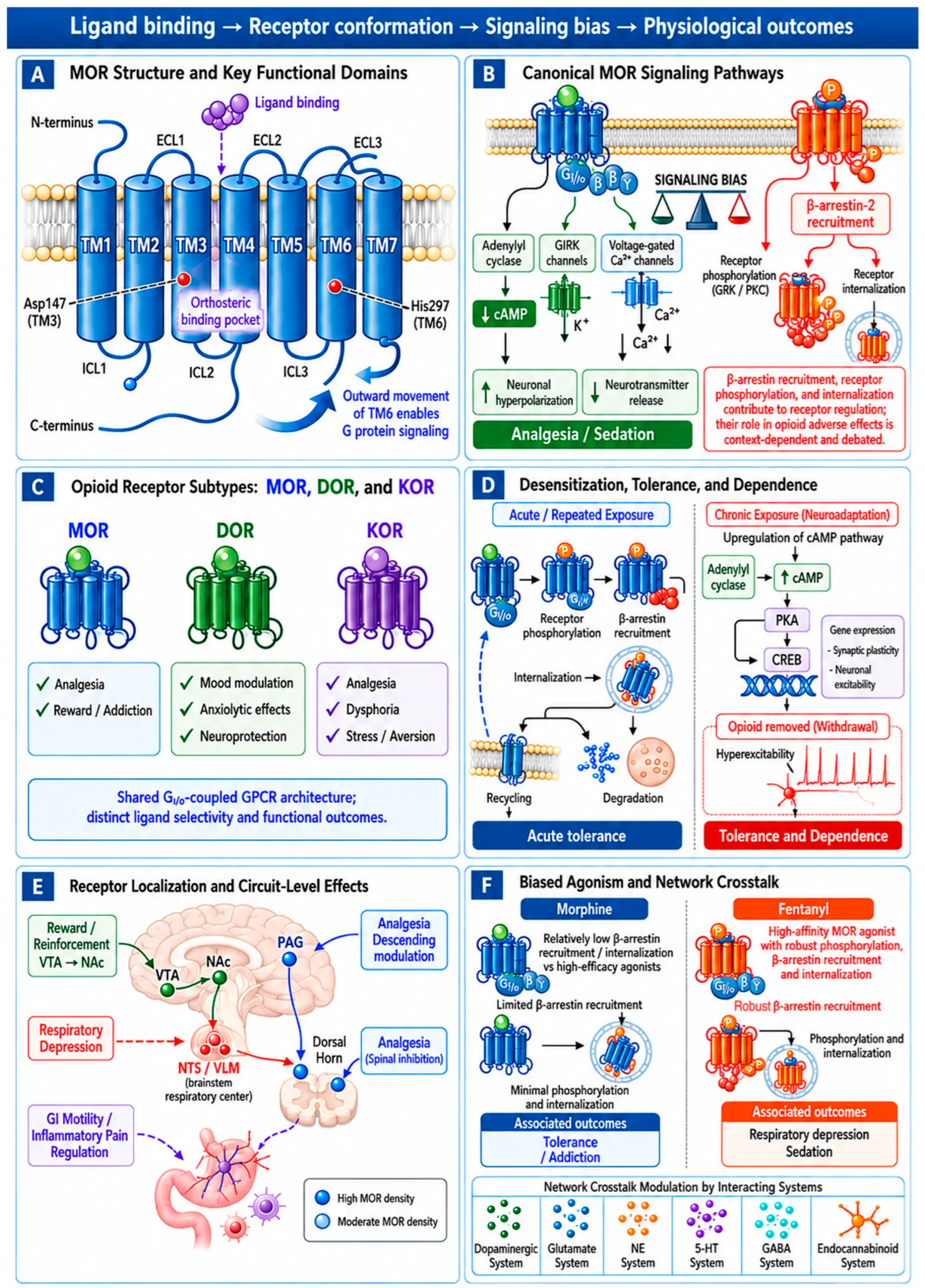
Ligand-dependent opioid receptor signaling bias and circuit-level outcomes. Opioid ligands bind to conserved GPCR structures, but stabilize distinct receptor conformations that shape downstream signaling, receptor regulation, and physiological effects. (**A**) Schematic of μ-opioid receptor (MOR) structure, highlighting the seven-transmembrane architecture, orthosteric ligand-binding pocket, intracellular loops, and conformational changes linked to G-protein activation. (**B**) Canonical MOR signaling includes G_i/o_-mediated inhibition of adenylyl cyclase, reduced cAMP signaling, activation of GIRK K^+^ channels, inhibition of voltage-gated Ca^2+^ channels, neuronal hyperpolarization, and reduced neurotransmitter release. GRK/PKC phosphorylation and β-arrestin recruitment contribute to receptor desensitization and internalization, although their roles in opioid adverse effects remain ligand-, cell-, and context-dependent. (**C**) MOR, DOR, and KOR share G_i/o_-coupled GPCR architecture, but differ in ligand selectivity, anatomical distribution, and functional outcomes. (**D**) Repeated or chronic opioid exposure drives receptor phosphorylation, β-arrestin recruitment, trafficking, recycling or degradation, and compensatory upregulation of cAMP/PKA/CREB signaling, contributing to tolerance, withdrawal-associated dependence, and altered neuronal excitability. (**E**) Circuit-level MOR localization links spinal and supraspinal antinociceptive pathways to analgesia, mesolimbic dopamine circuitry to reinforcement, and brainstem respiratory networks to respiratory depression, with additional modulation by gastrointestinal, inflammatory, and pain-regulatory systems. (**F**) Distinct ligands can differ in efficacy, phosphorylation patterns, β-arrestin recruitment, receptor trafficking, and network crosstalk, producing heterogeneous outcomes across analgesia, reinforcement/OUD risk, tolerance, dependence, and respiratory depression. Overall pathway engagement depends on ligand efficacy, dose, receptor reserve, receptor subtype, cell type, circuit context, exposure duration, and physiological state. Panel (**F**) illustrates selected ligand-associated outcomes rather than mutually exclusive effects: both morphine and fentanyl can produce tolerance, dependence, sedation and respiratory depression. Their different receptor-regulatory profiles do not by themselves establish the mechanism or magnitude of respiratory toxicity.

The MOR, encoded by the *oprm1* gene, belongs to the Class A (rhodopsin-like) G-protein-coupled receptor (GPCR) superfamily and represents a major molecular target through which endogenous opioid peptides and exogenous opioid drugs exert their effects [10,11]. Structurally, MOR consists of seven transmembrane α-helices (TM1–TM7) connected by three extracellular (ECL1–ECL3) and three intracellular loops (ICL1–ICL3), with an extracellular N-terminus and a cytoplasmic C-terminal tail (Figure 1A). This architecture enables translation of ligand binding into conformational changes that initiate intracellular signaling. The orthosteric binding pocket of MOR is formed by amino acid residues within transmembrane helices TM2, TM3, TM5, TM6, and TM7 [10]. Using mouse MOR residue numbering, Asp147 (TM3) anchors the protonated amine of many opioid ligands, while His297 (TM6) contributes to hydrogen-bonding interactions [12]. The engagement of ligands stabilizes the receptor’s active conformation that is characterized by an outward movement of TM6, exposing a cytoplasmic cavity for G-protein binding [12,13].

The MOR signals through two major pathways: G-protein-dependent and β-arrestin-mediated pathways. MOR couples to G_i/o_-type G-proteins, leading to inhibition of adenylyl cyclase and a decrease in intracellular cAMP [14] (Figure 1B). G_i/o_ signaling also promotes opening of G-protein-activated inwardly rectifying potassium (GIRK) channels and inhibition of voltage-gated calcium channels, leading to neuronal hyperpolarization and suppression of neurotransmitter release [15]. This signaling pathway controls MOR-mediated analgesic and sedative properties. In contrast, β-arrestin-2 mediates receptor desensitization, internalization, and MAPK pathway activation, specifically kinase cascades such as ERK and JNK [16]. Early studies associated β-arrestin recruitment with opioid adverse effects and motivated the development of G-protein-biased agonists [10,17,18]. Subsequent evidence indicates that respiratory depression is not universally β-arrestin-dependent; the limitations of this model are discussed in Section 2.5 [19]. The balance between these pathways is known as signaling bias and is a critical determinant in the pharmacological profile of opioids. The MOR N-terminal extracellular domain contains multiple N-linked glycosylation sites that support proper folding and membrane trafficking, consistent with GPCR biology [12]. However, the key functional domains that determine ligand binding and signaling are located primarily within the transmembrane helices and intracellular loops. The transmembrane helices form the structural core of the binding pocket, while ligand efficacy is determined by how ligand interactions propagate conformational changes to intracellular regions, particularly ICL3 [11]. The C-terminal tail contains serine and threonine residues acting as phosphorylation sites for GPCR kinases facilitating β-arrestin recruitment and receptor desensitization [20].

### 2.2. DOR and KOR

The κ-opioid receptor (KOR) and δ-opioid receptor (DOR) represent additional major members of the opioid receptor family, contributing to distinct physiological and pharmacological effects. Like MOR, both KOR and DOR are Class A GPCRs that couple to G_i/o_ proteins, leading to inhibition of adenylyl cyclase and reduction of neuronal excitability [21]. Activation of KOR produces analgesia, but is also associated with dysphoria, hallucinations, and stress-induced aversion, limiting therapeutic use. DOR activation has been implicated in mood modulation, anxiety, and emotional responses, as well as analgesia and neuroprotection [22]. Although these three opioid receptors share a conserved transmembrane architecture, differences in receptor structure contribute to ligand selectivity and pharmacological responses [21]. Understanding the interactions among MOR, KOR, and DOR signaling pathways is crucial in developing next-generation opioids that maximize analgesia while minimizing side effects and abuse potential (Figure 1C).

### 2.3. Tolerance, Dependence, and Receptor Desensitization

Repeated MOR activation can reduce responsiveness to opioid exposure, contributing to tolerance and an increased dose requirement for a given pharmacological effect. Receptor-level mechanisms include phosphorylation, β-arrestin recruitment, desensitization, and altered trafficking [14,23]. Phosphorylated receptors recruit β-arrestins, which can limit G-protein coupling; internalized receptors may recycle to the membrane or undergo degradation, depending on the ligand and cellular context [14,23]. Cellular studies further show that MOR C-terminal sequence elements and the surface lifetime of receptor–arrestin clusters regulate arrestin-mediated signaling, with distinct profiles for endomorphin-2 and morphine [24]. Physical dependence reflects adaptations that become apparent as withdrawal following opioid discontinuation or antagonism. These include compensatory changes in adenylyl cyclase, cAMP/PKA signaling, and CREB-mediated transcription that can produce withdrawal-associated hyperexcitability [25,26] (Figure 1D). The ligand-dependent receptor-regulatory differences underlying these processes are discussed in Section 2.5.

### 2.4. Receptor Localization

Opioid receptors are distributed throughout the central and peripheral nervous systems, with distinct patterns shaping their functional diversity and pharmacological outcome. MOR is expressed in the brainstem, particularly within the periaqueductal gray (PAG), nucleus tractus solitarius (NTS), and ventrolateral medulla (VLM), where it mediates potent analgesic and respiratory depressant effects [23,27]. In the mesolimbic reward circuitry, MOR is located in the GABAergic interneurons of the ventral tegmental area (VTA) and in the nucleus accumbens (NAc). MOR activation in the VTA disinhibits dopaminergic neurons and increases dopamine release in the NAc, which is a key mechanism driving reward, reinforcement, and addiction [28]. KOR displays a complementary distribution, enriched in the hypothalamus, amygdala, and nucleus accumbens shell, contributing to stress responsivity, dysphoria, and aversive motivational stress [29]. In contrast, the DOR is abundantly expressed in the neocortex, olfactory bulb, and striatum and, to a lesser extent, in limbic structures, where it modulates mood regulation, anxiety, and neuroprotection [22]. Opioid receptors are also located in the spinal cord, specifically within the dorsal horn and lamina II (substantia gelatinosa), where they suppress nociceptive transmission by inhibiting the release of excitatory neurotransmitters such as substance P and glutamate [30]. The activation of MOR and DOR is critical for spinal analgesia, while activation of KOR contributes to antinociceptive effects by modulating spinal GABAergic interneurons. In the peripheral nervous system, opioid receptors are expressed on the primary afferent neurons, immune cells, and enteric neurons, where they regulate inflammatory pain and GI motility [31]. Understanding region-specific localization provides critical insights into the mechanistic basis of opioid addiction and informs the design of targeted therapeutics aimed at dissociating analgesic efficacy from adverse effects and abuse potential (Figure 1E).

### 2.5. Biased Agonism Between Morphine and Fentanyl

Even though opioid agonists act through a common receptor framework, individual ligands can stabilize distinct MOR conformations and thereby differ in signaling efficacy, receptor regulation, and downstream cellular response. This phenomenon, commonly referred to as ligand bias or biased agonism, describes the preferential engagement of one signaling or regulatory pathway relative to another under defined experimental conditions. At MOR, particular attention has focused on differences between G-protein-mediated signaling and pathways involving receptor phosphorylation, β-arrestin recruitment, desensitization, and internalization. Importantly, signaling bias is not an intrinsic binary property of a ligand; its apparent magnitude can vary depending on receptor expression, receptor reserve, cellular background, assay system, ligand concentration, and the signaling endpoint measured [32,33]. In MOR-expressing cells, morphine and DAMGO produced different receptor distributions within the plasma membrane and distinct spatial and temporal ERK-signaling profiles, indicating that membrane organization can influence signaling independently of internalization [34].

Morphine and fentanyl illustrate these ligand-dependent differences. Both activate MOR and engage G_i/o_-dependent signaling, resulting in inhibition of adenylyl cyclase, reduced intracellular cAMP, modulation of GIRK potassium and voltage-gated calcium channels, and suppression of neurotransmitter release. However, their efficacy and receptor-regulatory profiles differ. Morphine generally produces strong G-protein activation, while inducing comparatively limited MOR phosphorylation, β-arrestin recruitment, and receptor internalization relative to high-efficacy agonists such as fentanyl [14,35]. Consequently, substantial MOR signaling can persist with relatively limited receptor trafficking. During repeated exposure, these properties occur together with compensatory adaptations in intracellular signaling, physical dependence, and withdrawal-associated neuronal hyperexcitability [23].

In contrast, fentanyl, a high-efficacy synthetic MOR agonist, produces robust G-protein activation together with pronounced receptor phosphorylation, β-arrestin recruitment, desensitization, and internalization [36]. These regulatory events can promote receptor trafficking, recycling, or degradation depending on the cellular context and duration of exposure. Therefore, differences between fentanyl and morphine extend beyond the relative activation of G-protein versus β-arrestin pathways and include ligand efficacy, receptor kinetics, phosphorylation patterns, trafficking behavior, pharmacokinetics, and the neuronal circuits in which MOR is expressed. These factors collectively contribute to differences in the onset, magnitude, and duration of analgesic, rewarding, sedative, and respiratory effects [3]. Early models of MOR-biased agonism proposed a relatively simple functional separation in which G-protein signaling primarily mediated analgesia, whereas β-arrestin-2 signaling was responsible for adverse effects such as respiratory depression, constipation, and tolerance. This hypothesis provided an important rationale for the development of G-protein-biased MOR agonists [17,18]. However, subsequent studies have challenged a strict separation between these pathways, particularly for opioid-induced respiratory depression [19]. β-arrestin recruitment clearly contributes to receptor desensitization, trafficking, and signal regulation, but its contribution to specific adverse effects appears to depend on the ligand, cellular environment, receptor reserve, dose, and neural circuit involved. Therefore, respiratory depression should not be considered a universally β-arrestin-dependent consequence of MOR activation.

The concept of biased agonism has substantially influenced opioid drug development. Compounds designed to favor G-protein signaling or exhibit reduced intrinsic efficacy have been investigated as strategies for maintaining analgesia while improving the therapeutic window [33]. The clinical and preclinical experience with these ligands indicates that signaling selectivity may modify adverse-effect profiles, but complete separation of analgesia from respiratory depression, tolerance, or abuse liability has not yet been achieved. Contemporary models increasingly interpret MOR pharmacology as the product of ligand efficacy, signaling preference, receptor regulation, pharmacokinetics, receptor reserve, and circuit-specific signaling, rather than a binary assignment of therapeutic effects to G-proteins and adverse effects to β-arrestins (Figure 1F).

### 2.6. Alternative Signaling Pathways

Opioid receptor signaling does not act alone. Instead, it intersects with multiple neurotransmitter systems that act together and shape the complex neurobiology of addiction, tolerance, and overdose. The most prominent systems are dopaminergic, glutamatergic, adrenergic, serotonergic, GABAergic, and endocannabinoid. Their modulation defines the transition from acute opioid reward to chronic dependence and withdrawal.

#### 2.6.1. Dopaminergic and Glutamatergic Systems

Opioids show powerful effects on the mesolimbic dopamine pathway, which is a central component of reward and reinforcement. Activation of MOR on GABAergic interneurons in the VTA inhibits GABA release, leading to disinhibition of dopaminergic neurons and enhanced dopamine release in the NAc [37,38]. This contributes to the reinforcing properties of opioids and drives drug-seeking behavior. Drug-induced changes in glutamatergic signaling and synaptic plasticity within reward-related circuits contribute to craving and context-dependent drug seeking [39,40]. Dopaminergic and glutamatergic adaptations are central to long-term neuroplastic changes that characterize addiction.

#### 2.6.2. Adrenergic and Serotonergic Systems

The adrenergic system acts through the locus coeruleus (LC), playing a key role in arousal, autonomic regulation, and withdrawal responses. Acute MOR activation inhibits LC firing, producing sedation and anxiolysis; however, chronic opioid use induces compensatory upregulation of cAMP-dependent noradrenergic signaling, resulting in rebound hyperexcitability during withdrawal [41]. Serotonergic receptors also participate in pain and respiratory modulation. In rats, 5-HT7 receptors were localized pre- and postsynaptically in the dorsal spinal cord [42], and selective 5-HT4-receptor activation counteracted fentanyl-induced respiratory depression without abolishing analgesia [43].

#### 2.6.3. GABAergic and Endocannabinoid Systems

Opioid-induced modulation of GABAergic neurotransmission is central to both analgesia and reward. MOR is located on presynaptic GABAergic terminals in the VTA and periaqueductal gray (PAG), where MOR activation inhibits GABA release, facilitating dopaminergic disinhibition and descending pain inhibition [44]. Chronic opioid use remodels inhibitory synapses, leading to altered GABA tone and contributing to tolerance and dependence. The endocannabinoid system (ECS) extensively overlaps with opioid circuits. Cannabinoid 1 (CB1) receptors are co-expressed with MOR in the NAc, amygdala, and PAG, engaging in bidirectional crosstalk through G_i/o_-coupled pathways. Interactions between cannabinoid and opioid systems modulate analgesia, reward, tolerance, and dependence [45].

#### 2.6.4. Integrated Perspective

These neuromodulator systems connect reward, stress responses, emotional regulation, and homeostasis. Opioid exposure modifies this network through direct receptor-mediated effects and changes in excitatory and inhibitory transmission. The consequences depend on the circuit, exposure history, and endpoint measured. This distinction is important when relating adaptations associated with repeated opioid use to the immediate respiratory toxicity of overdose.

Although opioid reward, tolerance, physical dependence, addiction, and overdose are mechanistically interconnected, they represent distinct biological phenomena. Reward and reinforcement reflect acute motivational effects that can promote repeated opioid use, whereas tolerance and physical dependence develop through adaptive cellular and circuit-level changes during repeated exposure. Opioid use disorder involves persistent alterations in motivation, behavioral control, cue responsiveness, and relapse vulnerability and should not be considered synonymous with either tolerance or physical dependence. Acute opioid overdose is primarily a toxic state resulting from excessive MOR-mediated suppression of respiratory and arousal networks. Chronic neuroadaptations can modify susceptibility to overdose, but are mechanistically distinct from the immediate processes responsible for acute respiratory toxicity.

## 3. Opioid Addiction Neurocircuit: Reward, Tolerance, and Dependence

This section focuses on processes associated with repeated opioid exposure and the development of addiction-related phenotypes, including reward, reinforcement, tolerance, physical dependence and persistent changes that contribute to compulsive drug use and relapse. These adaptations are distinguished from acute respiratory and arousal suppression responsible for opioid overdose, which is discussed separately in Section 4 and Section 5.

### 3.1. Mesolimbic Dopamine Signaling in Opioid Reward and Reinforcement

The mesolimbic dopamine (DA) pathway forms the central neurobiological substrate of opioid reward and reinforcement. Originating in the VTA and projecting to the NAc, this circuit integrates dopaminergic signaling with glutamatergic, GABAergic, serotonergic, and endocannabinoid modulation to encode the motivational and hedonic properties of opioids [46,47]. Under physiological conditions, VTA dopaminergic neurons are tightly regulated by local GABAergic interneurons. MOR activation hyperpolarizes inhibitory interneurons and reduces GABAergic input to DA neurons, thereby producing disinhibition [37]. The resulting increased DA release in the NAc mediates the euphoric “high” associated with opioid use and reinforces drug-seeking behavior [48]. Ligand efficacy, pharmacokinetics, and circuit engagement can modify opioid reward profiles. The receptor-regulatory differences between morphine and fentanyl described in Section 2.5 do not, by themselves, establish the magnitude or duration of reinforcement or the rate of tolerance development [14,15,36]. Interactions with glutamatergic inputs from the prefrontal cortex (PFC) and amygdala further shape reward encoding. N-methyl-D-aspartate (NMDA) receptor-dependent plasticity in the VTA and NAc contributes to the transition from acute reward to chronic drug seeking [39]. In addition, cannabinoid–opioid interactions modulate reward-related signaling and dependence [45]. Endogenous enkephalin–MOR signaling in a dorsal-raphe-to-nucleus-accumbens circuit also regulates state-dependent reward consumption in mice, illustrating the circuit specificity of physiological opioid signaling [49]. These overlapping networks create a dynamic feedback system that fine-tunes the balance between hedonic drive and inhibitory control, underpinning the compulsive nature of opioid addiction.

### 3.2. Prefrontal Cortex

The prefrontal cortex (PFC) plays a pivotal role in executive control, decision-making, emotional regulation, and inhibitory control over reward-driven behaviors. In the context of opioid addiction, the PFC serves as the principal top-down regulator of subcortical regions such as the nucleus accumbens (NAc), amygdala, and ventral tegmental area (VTA). Altered PFC function is associated with impaired self-control, impulsivity, and compulsive drug seeking in addiction [50,51]. Repeated drug exposure can produce synaptic remodeling in addiction-related circuits, including changes in glutamate receptor function and synaptic plasticity [40]. Dopaminergic and serotonergic modulation of corticostriatal circuits also contributes to impulse control and addiction-related behavior [51]. Human neuroimaging studies implicate altered PFC function in addiction, including disrupted executive control and regulation of limbic reward regions [50]. These deficits contribute to decision-making impairments, heightened cravings, and relapse vulnerability. Collectively, the PFC functions as the neurobiological “brake” in the addiction circuitry. PFC dysfunction can weaken executive control over reward-driven behavior and contribute to compulsive drug seeking.

### 3.3. Amygdala and Hippocampus

The amygdala and hippocampus are key limbic structures that integrate emotional valence, contextual memory, and stress reactivity in opioid addiction. Together, they encode the emotional salience of drug-associated cues and the contextual memories that trigger craving and relapse [46]. Within this circuitry, the amygdala assigns affective meaning to opioid-related stimuli, while the hippocampus contextualizes these experiences by linking drug exposure to specific environments [52,53]. Opioids strongly modulate amygdala circuits, particularly within the central amygdala (CeA) and basolateral amygdala (BLA). During opioid withdrawal, CRF (corticotropin-releasing factor) and noradrenergic signaling within the extended amygdala contribute to negative affect and withdrawal aversion [54]. This anti-reward state sustains compulsive use by shifting motivation from positive reinforcement to avoiding dysphoria. The hippocampus contributes to the contextual learning and memory processes that shape drug seeking [53]. In adult rats, chronic morphine administration and heroin self-administration reduced hippocampal neurogenesis [55]. During withdrawal or abstinence, hippocampal activation enhances cue-induced craving by reactivating opioid-associated memory traces. These limbic neuroadaptations form the emotional core of addiction, linking stress, memory, and reward. Dysregulation of amygdala-hippocampal networks promotes persistent craving and relapse vulnerability, making them promising targets for interventions aimed at disrupting cue–context associations in opioid use disorder.

### 3.4. Neuroadaptations

Chronic opioid exposure induces extensive changes and neuroadaptations across multiple brain regions, reshaping synaptic connectivity, receptor function, and intracellular signaling to sustain addiction. At the cellular level, repeated MOR activation leads to homeostatic compensations, including downregulation of receptor density, reduced G-protein-coupling efficiency, and upregulation of the cAMP–PKA pathway, which collectively diminish acute drug responsiveness and contribute to tolerance [14,56]. In mouse striatal medium spiny neurons, acute and repeated morphine exposure produced cell-type-specific adaptations in dopamine-evoked cAMP signaling, with distinct responses in D1- and D2-receptor-expressing populations [57]. More broadly, opioid-related neuroadaptations occur within the VTA, NAc, amygdala, and prefrontal cortex, where synaptic plasticity mechanisms such as long-term potentiation (LTP) and long-term depression (LTD) are altered, reshaping the excitatory–inhibitory balance that governs reward and aversion [40,46]. Neuroadaptations extend beyond dopamine circuits. Chronic opioid exposure disrupts glutamatergic signaling by altering AMPA/NMDA receptor expression and trafficking in the NAc and PFC, facilitating drug-cue association learning [39]. Additionally, opioids influence GABAergic, serotonergic, noradrenergic, and endocannabinoid systems, modulating stress responses, affective states, and withdrawal symptoms [45,54]. Together, these multi-system adaptations create a persistent drug-dependent state, characterized by heightened reward sensitivity, negative affect during withdrawal, and vulnerability to relapse.

### 3.5. Receptor and Downstream Adaptations Underlying Opioid Tolerance and Dependence 

Repeated opioid exposure shifts dose–response relationships through receptor-level and downstream adaptations. MOR desensitization and trafficking contribute, with the magnitude and direction of regulation depending on the ligand and cellular context described in Section 2.5. Compensatory changes in adenylyl cyclase, cAMP-dependent protein kinase activity, and ion-channel function further alter neuronal responses [14,56]. These processes contribute to tolerance, while the adaptations expressed during opioid discontinuation contribute to physical dependence and withdrawal. The presence of either does not, by itself, establish opioid use disorder.

### 3.6. Epigenetic Regulation and Long-Term Plasticity in Opioid Addiction

Chronic opioid exposure produces persistent epigenetic modifications that reshape gene expression and neuronal plasticity, contributing to the long-lasting nature of addiction. These modifications include DNA methylation, histone acetylation and methylation, and regulation by microRNAs (miRNAs), which collectively influence transcription of genes involved in synaptic transmission, plasticity, and stress response [58,59]. Key transcription factors, such as CREB and ΔFosB, are upregulated in reward-related regions—including the nucleus accumbens, prefrontal cortex, and amygdala—following repeated opioid exposure. CREB activation modulates cAMP-dependent pathways and contributes to tolerance and withdrawal-associated dysphoria, whereas ΔFosB accumulates gradually and persists for weeks, stabilizing synaptic adaptations that reinforce drug-seeking behavior [60,61]. Opioid-induced epigenetic changes also alter glutamatergic and GABAergic transmission, modifying AMPA/NMDA receptor composition and dendritic spine morphology, which strengthens drug-cue associations and conditioned responses. Additionally, miRNA-mediated regulation can fine-tune MOR signaling and downstream effectors, further contributing to long-term plasticity in mesolimbic circuits [62]. These epigenetic mechanisms help explain why addiction persists beyond detoxification, making them promising targets for therapeutic interventions aimed at reversing maladaptive neuronal remodeling.

### 3.7. Zebrafish Insights: CPP, LMA, CRISPR

**Figure 2:**
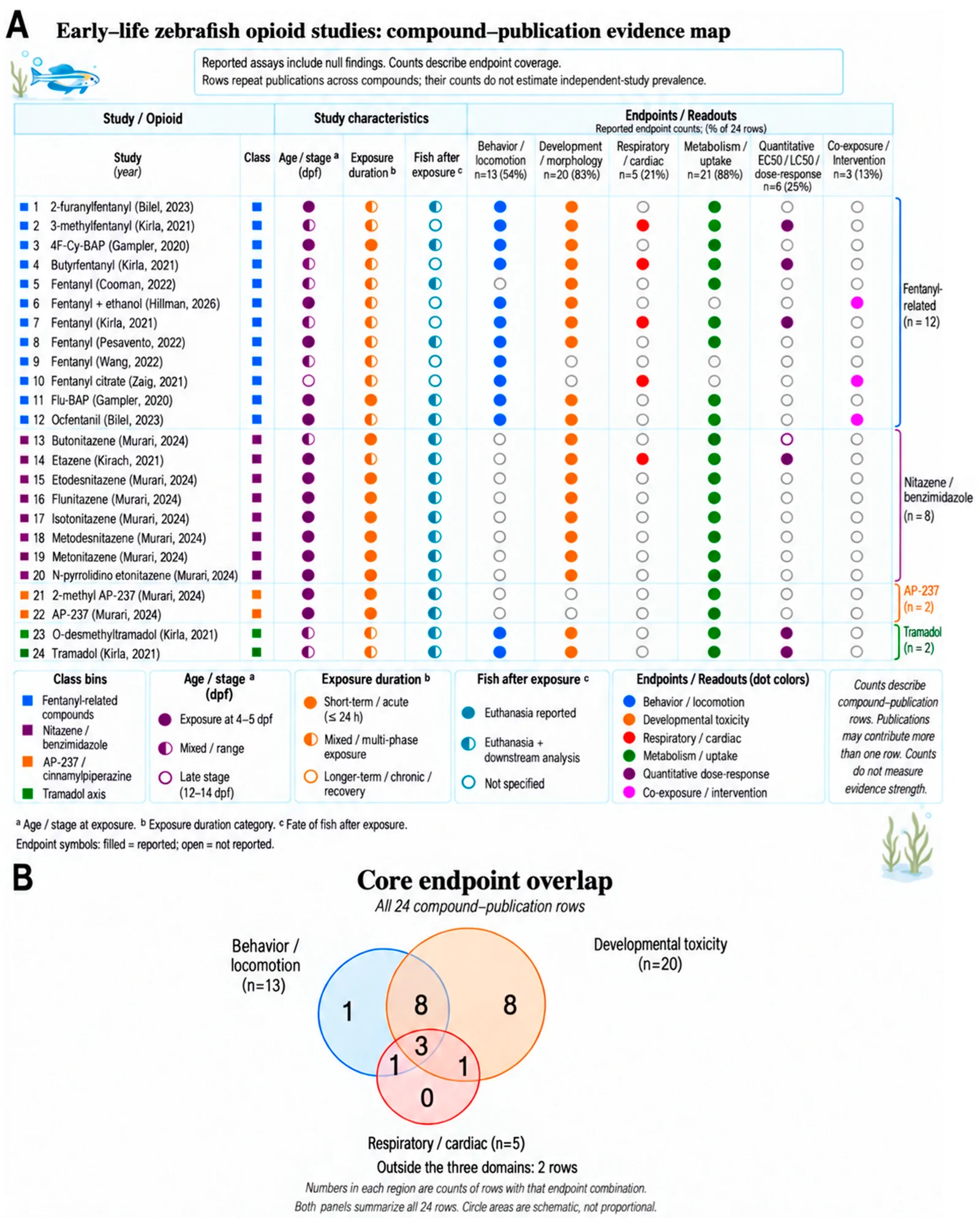
Compound–publication evidence map of opioid exposure in zebrafish embryos and larvae. (**A**) The 24 rows represent opioid–publication pairings from 10 papers [5,7,9,67,68,69,70,71,72,73], not independent studies. Filled endpoint symbols denote a reported endpoint or analysis; open symbols denote an endpoint not reported in the matrix. A performed assay is counted even when its findings are null. Header counts and percentages use all 24 rows. Metabolism/uptake covers experimental or modeled uptake and metabolic analyses; the quantitative category covers reported EC50/LC50 or dose–response analyses. These broad categories do not imply identical methods across studies. Cardiac observations and respiratory-related readouts share one descriptive column, but are not equivalent measurements. In particular, mandibular movements [5] are not mammalian ventilation. (**B**) The Venn diagram shows the number of rows in each combination of the three core domains: 13 behavioral, 20 developmental, and 5 respiratory/cardiac entries. Two rows fall outside all three domains. Circle areas are schematic. Repeated publications and differences in assays preclude interpreting these counts as evidence strength or human-risk estimates. dpf, days post-fertilization; EC50, half-maximal effective concentration; LC50, median lethal concentration.

Zebrafish provide complementary behavioral and molecular readouts of opioid exposure. Conditioned place preference (CPP) measures learned preference for a drug-associated environment; it should be interpreted as a reward-related phenotype rather than a direct measure of addiction. Fentanyl-associated CPP has been reported in adult zebrafish [63], although the predictive value of zebrafish CPP for human abuse potential remains limited and depends on the assay and compound class [64]. An adult zebrafish opioid self-administration assay provides a complementary measure of drug-seeking behavior and enables pharmacological interrogation of the pathways supporting opioid reinforcement [65]. Locomotor assays measure changes in activity that may reflect stimulation, sedation, or toxicity and require separate controls to distinguish these effects. Molecular studies provide additional evidence of chronic exposure: zebrafish brain proteomics identified protein changes following repeated tramadol administration [66]. CRISPR-based disruption of *oprm1* could test receptor-specific contributions to these phenotypes, but this requires a validated zebrafish model. The methodological rationale for future zebrafish opioid-receptor models is discussed in Section 5.7. Figure 2 summarizes compound–publication entries for opioid exposure in zebrafish embryos and larvae. Clearly, the studies summarized in Figure 2 report no co-exposure or intervention assays for recently emerging synthetic opioids, such as nitazenes, with these approaches confined to fentanyl-related entries. Despite the high-throughput potential of zebrafish, only 6 of 24 entries (25%), including 3 of 12 fentanyl-related entries, report EC50/LC50 estimates or quantitative dose–response analyses. Respiratory-related or cardiac readouts are similarly uncommon (5/24, 21%), highlighting opportunities in the published literature for rigorous dose–response profiling and for complementary physiological assays to strengthen interpretation of behavioral and developmental endpoints.

## 4. Opioid Overdose Neurocircuit: Acute Toxicity and Risk Modifiers

Acute opioid overdose is mechanistically distinct from the chronic neuroadaptations underlying tolerance, dependence, and addiction. Whereas repeated opioid exposure progressively alters receptor signaling, synaptic plasticity, and reward-related circuitry, acute overdose is dominated by excessive MOR activation and rapid suppression of neural networks required for respiration, arousal, and autonomic homeostasis. Pre-existing tolerance and other chronic adaptations can alter opioid sensitivity and overdose vulnerability; however, these processes should be considered modifiers of acute toxicity rather than the immediate mechanisms producing overdose. The following sections therefore focus on circuit-level dysfunction occurring during acute high-dose opioid exposure and its relationship to respiratory failure.

### 4.1. Mesolimbic Circuitry as an Indirect Modifier of Overdose Risk

Mesolimbic circuitry contributes to opioid reinforcement and repeated drug seeking, thereby influencing the probability and pattern of exposure [46,47]. These behavioral effects should be distinguished from the brainstem and arousal-network suppression that produces acute overdose. During intoxication, MOR activation alters inhibitory transmission in reward circuits, but changes in mesolimbic dopamine alone do not establish the mechanism of respiratory failure [14,56]. The distinction separates circuit-level influences on exposure and vulnerability from the direct respiratory effects discussed in Section 5.

### 4.2. Prefrontal Cortex in Overdose

The prefrontal cortex (PFC) supports executive function, decision-making, and inhibitory control over drug-seeking behavior [50,51]. PFC dysfunction associated with addiction can influence the choices and patterns of exposure that contribute to overdose vulnerability. These findings concern behavioral risk and do not establish PFC suppression as a direct cause of acute respiratory failure. The respiratory-control mechanisms of opioid toxicity are discussed in Section 5.

### 4.3. Amygdala and Hippocampus in Overdose

Amygdala and hippocampal circuits integrate stress, emotional responses, and contextual memory, influencing drug seeking and relapse [46,52,53]. Amygdala stress systems contribute to withdrawal-associated negative affect and negative reinforcement [54], while chronic morphine administration and heroin self-administration reduced hippocampal neurogenesis in adult rats [55]. These findings help explain how exposure history and learned associations may influence future high-risk exposure. They do not establish that limbic dysregulation directly produces the acute respiratory failure of opioid overdose.

### 4.4. Neuroadaptations in Overdose

Chronic opioid-induced neuroadaptations can modify the response to subsequent exposure without constituting the acute mechanism of overdose. Receptor regulation, compensatory cAMP/PKA signaling, and synaptic plasticity develop with repeated use [14,56]. Their consequences depend on the affected circuit and the endpoint used to assess tolerance. Accordingly, tolerance to one opioid effect cannot be assumed to confer equivalent protection against respiratory suppression. Acute toxicity remains dependent on the dose and time course of MOR activation in respiratory and arousal networks. Chronic adaptations should therefore be treated as potential modifiers of this response rather than evidence that reward-circuit remodeling directly causes respiratory failure.

### 4.5. Uneven Tolerance Across Opioid Effects and Residual Overdose Risk

Opioid tolerance modifies but does not eliminate overdose risk. As described in Section 2.3 and Section 3.5, chronic exposure produces receptor-level and downstream adaptations that reduce sensitivity to some opioid effects. However, tolerance does not necessarily develop uniformly across analgesic, rewarding, sedative, and respiratory effects. Hence, exposure to high-potency opioids can still overwhelm compensatory mechanisms and profoundly suppress respiratory and arousal circuits. Overdose vulnerability reflects the interaction among prior opioid exposure, degree of tolerance, opioid potency and dose, pharmacokinetics, and the sensitivity of respiratory-control networks [14,56].

### 4.6. Persistent Epigenetic Adaptations: Potential Relevance to Overdose Risk

Chronic opioid exposure can produce persistent changes in DNA methylation, histone modification, and microRNA-mediated regulation that influence neuronal function [47,59]. CREB and ΔFosB contribute to transcriptional adaptations associated with repeated exposure, withdrawal, and drug seeking [60,61], while microRNAs can regulate MOR expression [62]. These findings provide a basis for studying how exposure history modifies subsequent opioid responses. They do not, by themselves, demonstrate that a particular epigenetic change causes acute respiratory depression or explains overdose after abstinence. Establishing that relationship requires direct measurements in respiratory-control circuits and separation of persistent molecular effects from changes in tolerance and drug exposure.

### 4.7. Zebrafish Insights: CPP, Locomotor Activity, and CRISPR Models

**Figure 3:**
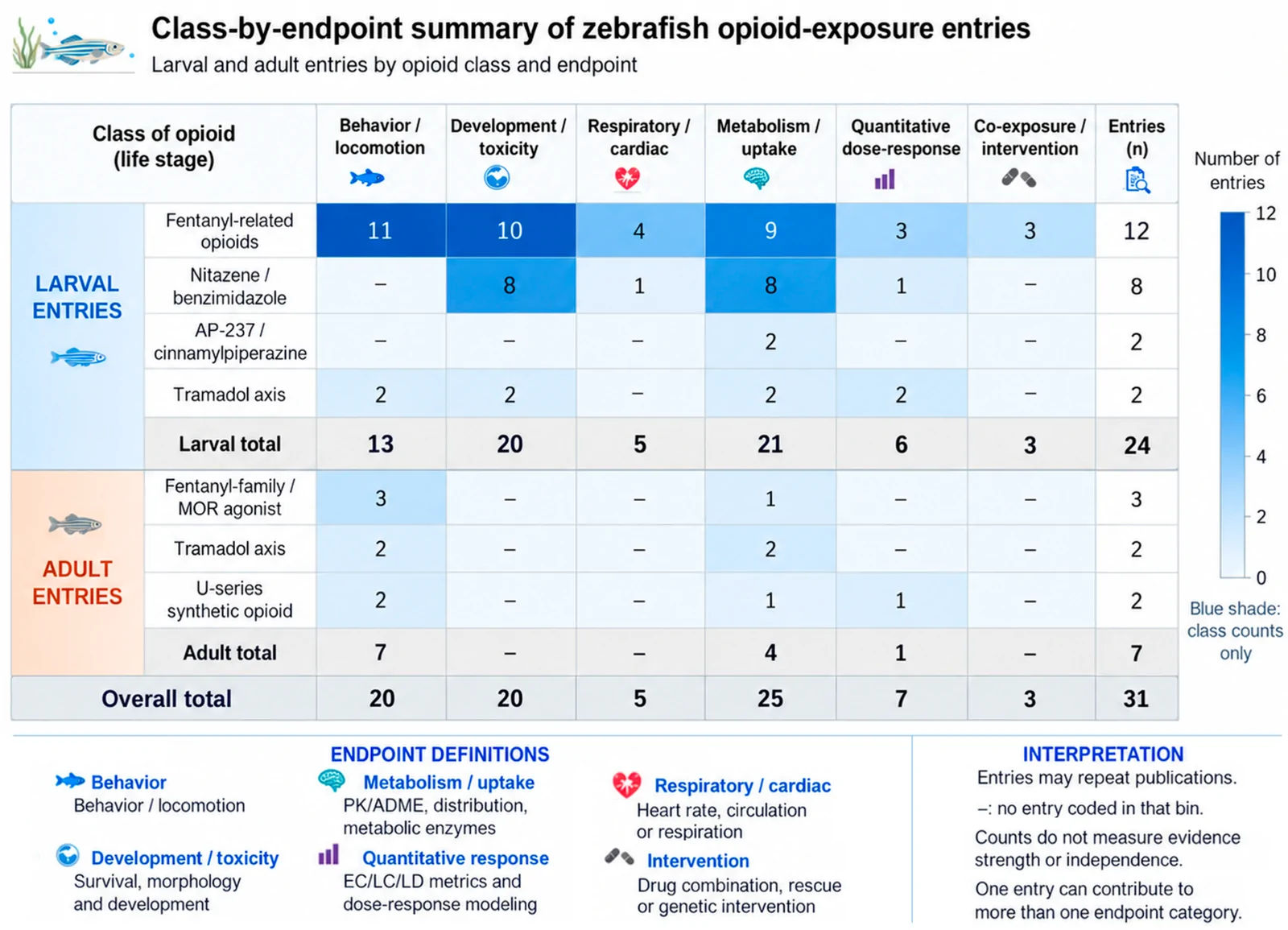
Class-by-endpoint overview of zebrafish opioid-exposure entries. The matrix summarizes 24 larval entries from Figure 2 and seven adult entries, with 31 entries overall. A reported assay is counted even when its findings are null. Counts indicate entries assigned to each endpoint category; they are not counts of independent publications, and an entry may contribute to more than one category. Adult opioid-exposure entries include fentanyl-related behavior [63], U-series behavioral assays [74,75], and tramadol proteomics or pharmacokinetics [66,76]. The overall counts are 20 behavior/locomotion, 20 development/toxicity, 5 respiratory/cardiac, 25 metabolism/uptake, 7 quantitative dose–response and 3 co-exposure/intervention entries. Blue shading indicates class-level entry counts. A zero or dash indicates that no entry was coded in that category and does not indicate biological absence. Behavioral, cardiac, respiratory-related and analytical readouts remain distinct. Summary counts describe the selected literature and do not measure comparative evidence strength or human-risk prevalence. MOR, μ-opioid receptor; PK, pharmacokinetics; ADME, absorption, distribution, metabolism and excretion; EC, effective concentration; LC, lethal concentration; LD, lethal dose.

Zebrafish assays can distinguish reward-related behavior from sedation and other toxic effects, provided that each endpoint is measured and interpreted separately. CPP addresses learned drug preference, whereas locomotor activity measures behavioral output; reduced movement alone does not demonstrate ventilatory depression. Respiratory-related measurements, including mandibular movements in larvae, require separate assessment and retain the physiological limitations discussed in Section 5.7 [5]. Validated *oprm1* loss-of-function models could help establish receptor dependence. The general GPCR genome-editing evidence summarized in Section 5.7 does not establish that MOR-knockout zebrafish show reduced CPP or protection from overdose; those outcomes require direct testing in a validated receptor-specific model. Figure 3 presents a class-level overview of larval and adult opioid-exposure endpoints; its quantitative limitations are stated in the legend. Of note from this analysis is the paucity of studies on fentanyl-type synthetic opioids in adult fish, including no reports of quantitative dose response studies, co-exposure/overdose reversal, or study of cardiac and respiratory function.

## 5. Opioid Overdose Neurobiology: Respiratory Depression and Fatal Outcomes

### 5.1. Brainstem Respiratory Centers (preBötzinger Complex)

**Figure 4:**
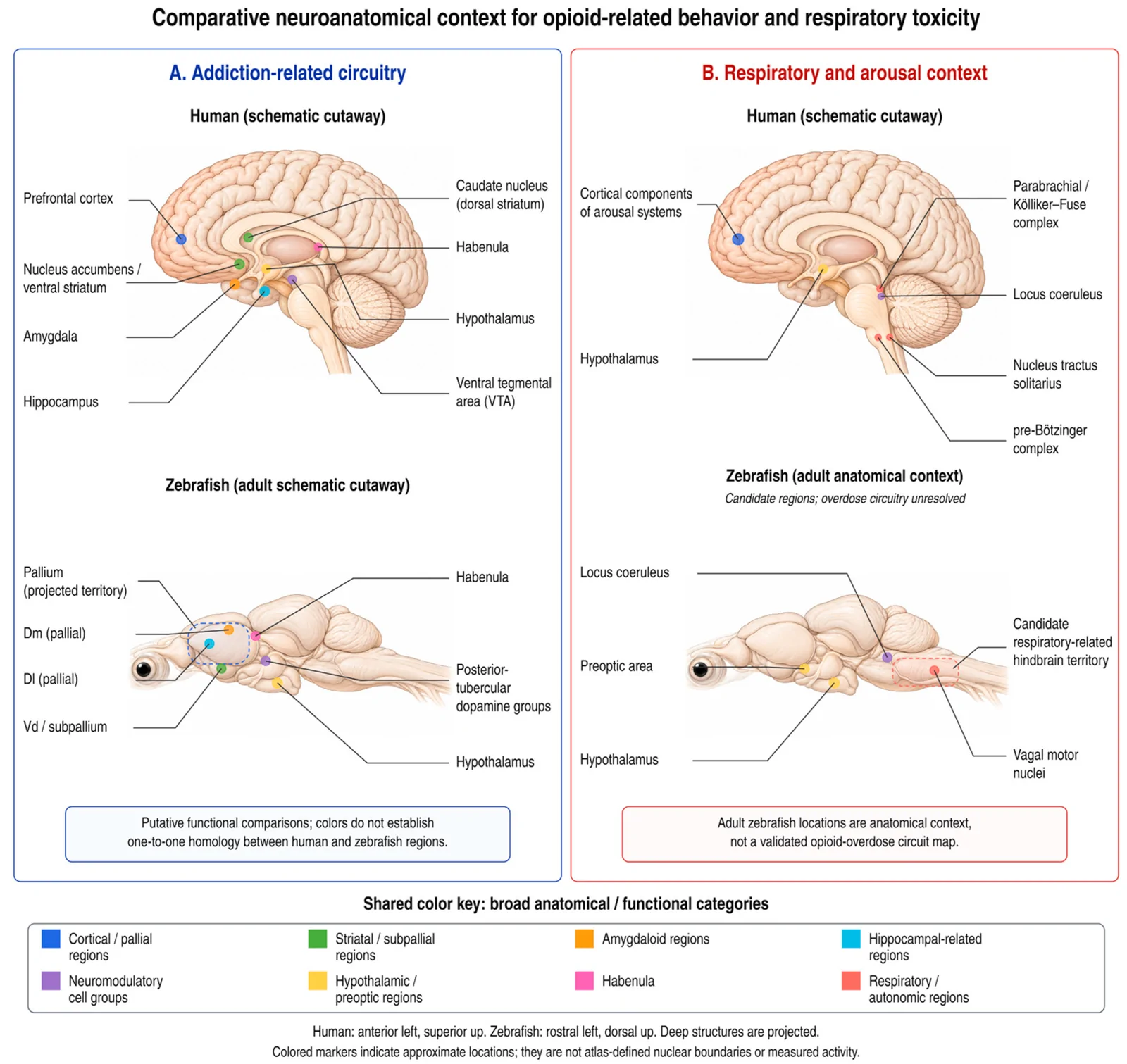
Comparative neuroanatomical context for opioid-related behavior and respiratory toxicity. Schematic cutaway projections show approximate locations: in the human views, anterior is left and superior is up; in zebrafish, rostral is left and dorsal is up. Deep structures are projected into the view, and markers do not delineate nuclear boundaries. Locations are informed by adult zebrafish atlases and human anatomical studies [82,83,84,85,86]. (**A**) Human addiction-related regions are compared with putatively corresponding zebrafish territories. Dm and Dl lie within the zebrafish pallium, distinct from subpallial Vd; their amygdaloid- and hippocampal-related interpretations remain qualified [87]. Posterior-tubercular dopamine groups provide an ascending system to the basal telencephalon [88]; their inclusion here does not imply one-to-one equivalence with mammalian midbrain dopamine nuclei. (**B**) Human respiratory/arousal sites are shown alongside adult zebrafish anatomical regions proposed for further study. The adult fish panel is anatomical in context, not a validated opioid-overdose circuit map; its dashed hindbrain territory is intentionally approximate. Opioid-sensitive mandibular-movement findings in larvae [5] do not establish the adult regional assignments. Colors organize broad anatomical/functional categories, not direct homology, measured activation or equivalent physiological outcomes.

Opioid overdose impairs this network, leading to life-threatening respiratory depression, which is the primary cause of mortality in acute opioid toxicity (Figure 4).The preBötzinger complex (preBötC) in the ventrolateral medulla is a critical pacemaker for generating rhythmic respiratory activity. It contains a network of excitatory and inhibitory neurons that drive inspiratory rhythm and coordinate downstream motor outputs to respiratory muscles [77,78]. Opioid overdose impairs this network, leading to life-threatening respiratory depression, which is the primary cause of mortality in acute opioid toxicity. MORs are densely expressed in the preBötC, where their activation hyperpolarizes neurons via G protein-mediated opening of potassium channels (GIRKs) and suppression of voltage-gated calcium channels, reducing excitatory drive to the respiratory rhythm generator [79,80]. High-potency opioids, such as fentanyl, produce profound inhibition of preBötC output, leading to decreased inspiratory amplitude and frequency, hypoventilation, and eventual hypoxia. The preBötC also integrates inputs from chemo-sensitive neurons that detect CO_2_/pH changes and modulate ventilation. Opioid-mediated suppression of these feedback mechanisms diminishes compensatory responses to hypercapnia, exacerbating the risk of fatal respiratory arrest [81]. Understanding the cellular and circuit-level mechanisms of preBötC inhibition is critical for designing therapeutic strategies, including respiratory stimulants and approaches that limit MOR-mediated respiratory-network inhibition, to mitigate overdose lethality.

### 5.2. MOR Activation and Suppression

MOR activation suppresses respiratory-network activity through G_i/o_-dependent mechanisms, including GIRK channel activation, inhibition of voltage-gated calcium channels, and reduced presynaptic neurotransmitter release [80]. In brainstem respiratory circuits, these effects reduce excitatory drive and can decrease respiratory rate and tidal volume. The magnitude and time course of suppression depend on opioid efficacy, dose, pharmacokinetics, and route of administration. Inhibition of chemosensitive and premotor pathways also limits compensatory responses to hypercapnia and hypoxia [14]. Thus, G-protein signaling contributes directly to respiratory toxicity as well as analgesia; preferential G-protein engagement alone cannot be taken as evidence that an agonist will preserve respiratory drive.

### 5.3. Interaction of Serotonergic and Adrenergic Systems

Opioid-induced respiratory depression is modulated not only by direct MOR signaling but also by interactions with serotonergic and adrenergic systems in the brainstem. Serotonergic neurons, particularly in the raphe nuclei, provide excitatory drive to respiratory centers, including the preBötC and motor neurons controlling the diaphragm. Opioid-mediated inhibition of these neurons decreases serotonin release, reducing excitatory input and further slowing respiratory rhythm [79,89]. Similarly, adrenergic neurons in the locus coeruleus contribute to arousal and respiratory drive via noradrenaline release. MOR activation suppresses noradrenergic signaling, blunting ventilatory responsiveness to hypercapnia and hypoxia [80]. Suppression of these modulatory inputs may contribute to opioid-induced respiratory depression alongside direct inhibition of respiratory networks. Dysregulation of these systems contributes to interindividual variability in overdose susceptibility, as baseline serotonergic and adrenergic tone can influence compensatory capacity. Understanding these modulatory interactions provides mechanistic insight into why some individuals experience fatal respiratory depression at lower opioid doses and highlights potential therapeutic strategies, such as serotonergic or adrenergic agonists, to restore respiratory drive during opioid overdose.

### 5.4. Risk Factors

Opioid overdose risk is influenced by a combination of genetic, pharmacological, physiological, and environmental factors. Genetic variability in the μ-opioid receptor gene (*oprm1*), β-arrestin signaling, and cytochrome P450 enzymes (e.g., CYP2D6, CYP3A4) can alter opioid metabolism, receptor sensitivity, and signaling bias, affecting individual susceptibility to respiratory depression [90,91]. Pharmacological factors include opioid potency, formulation, and co-administration with sedatives, such as benzodiazepines or alcohol, which synergistically enhance central respiratory depression [47]. High-potency opioids like fentanyl or illicit analogs dramatically increase overdose risk due to rapid, profound MOR activation and a limited therapeutic window. Physiological factors include age, sex, body mass, pre-existing respiratory or cardiovascular conditions, and chronic opioid tolerance, which can unpredictably modify the dose–response relationship. Environmental and behavioral factors, such as polydrug use, high-dose self-administration, and low social support, further compound risk. A comprehensive understanding of these factors is critical for identifying high-risk populations, guiding harm-reduction strategies, and developing individualized interventions to prevent fatal outcomes.

### 5.5. Role of β-Arrestin and G-Protein-Biased Signaling

The original β-arrestin hypothesis was supported by studies reporting attenuated morphine-induced respiratory suppression in β-arrestin-2 knockout mice [18]. This observation helped motivate efforts to favor MOR G-protein signaling over β-arrestin recruitment. Subsequent experiments found that germline β-arrestin-2 deletion did not attenuate opioid-induced respiratory depression [19], limiting a general assignment of analgesia to G proteins and respiratory toxicity to β-arrestins. G_i/o_-dependent inhibition of adenylyl cyclase, modulation of ion channels, and suppression of neurotransmitter release occur in respiratory-control networks as well as analgesic circuits. β-Arrestin recruitment remains important for receptor regulation, but its contribution to a particular physiological effect requires ligand-, cell-, and circuit-specific evidence. Bias measurements may inform drug development [33]; they should be interpreted alongside intrinsic efficacy, receptor reserve, exposure, and direct respiratory measurements rather than treated as a surrogate for respiratory safety.

### 5.6. Fentanyl-Specific Dynamics

Fentanyl is a synthetic, high-potency μ-opioid receptor agonist with unique pharmacokinetic and pharmacodynamic properties that contribute to its high overdose potential. Its rapid brain penetration and strong MOR affinity result in abrupt, profound receptor activation, producing intense analgesia and reward, but also severe respiratory depression within minutes of administration [47,92]. Fentanyl can strongly inhibit respiratory-control networks, but its receptor phosphorylation and β-arrestin recruitment profile alone does not establish the mechanism or severity of respiratory suppression. The relevant determinants include efficacy, concentration at the receptor, and the neuronal circuits engaged [14,15]. The therapeutic window must be established from the relationship between effective and toxic exposures rather than inferred from potency alone. Co-administration with other central nervous system depressants, such as benzodiazepines or alcohol, further amplifies its toxic effects [47]. Understanding fentanyl’s distinct receptor kinetics, signaling bias, and brainstem targeting is essential for developing overdose mitigation strategies, including rapid-acting antagonists (e.g., naloxone) and candidate analgesics evaluated directly for respiratory effects.

### 5.7. Zebrafish Models of Oxygen Deprivation

Zebrafish provide a complementary system for studying opioid-sensitive behavioral, cardiovascular, hypoxia-response, and respiratory-related phenotypes. Larval gas exchange relies substantially on cutaneous respiration, which limits direct comparison with mammalian pulmonary ventilation. Reduced locomotion, sedation, bradycardia, and responses to hypoxia are distinct endpoints; none should be used alone as evidence of ventilatory depression. More specific respiratory-related measurements are possible. Zaig et al. measured mandibular movements in freely behaving larvae and reported fentanyl-induced suppression that was reversed by naloxone [5]. This assay provides a readout of opioid-sensitive respiratory-related motor activity while remaining physiologically distinct from mammalian ventilation. Its interpretation should therefore be tied to developmental stage, experimental conditions, and the measured endpoint.

**Figure 5:**
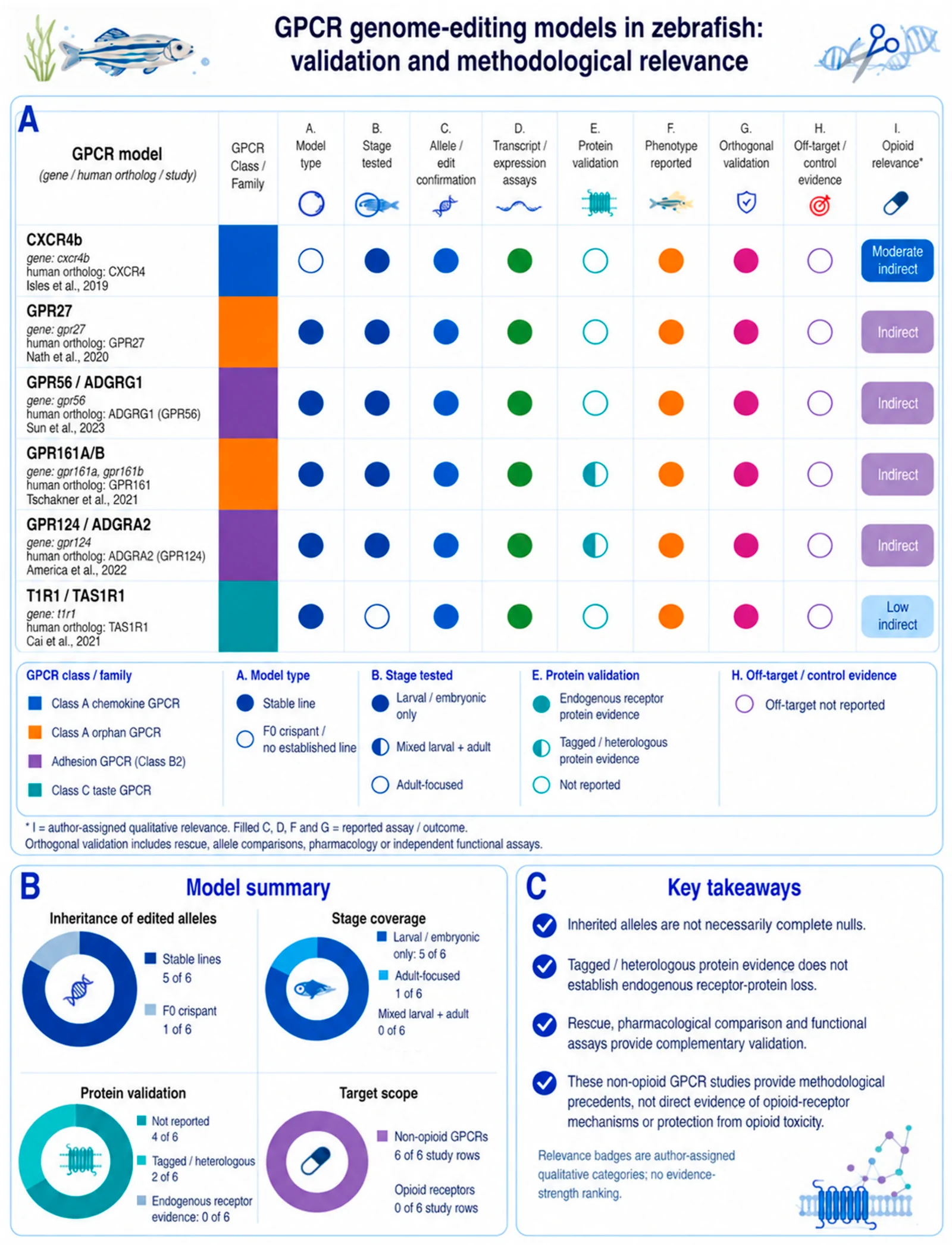
Validation characteristics and methodological relevance of GPCR genome-editing models in zebrafish. (**A**) Six study/model rows summarize non-opioid GPCR examples [94,95,96,97,98,99], with *gpr161a*/*gpr161b* grouped in one row. The Gpr161 study is Tschaikner et al. (2021) [97]; the Gpr124 study is America et al. (2022) [98], and its human ortholog is ADGRA2. The *cxcr4b* study used F0 crispants [94], while inherited alleles include Gpr124 intracellular-domain truncations [98]; inheritance does not establish a complete null. Transcript/expression assays are distinct from endogenous receptor-protein loss. (**B**) Summary counts follow the matrix classifications: five stable-line rows and one F0-crispant row; four larval/embryonic, one mixed larval/adult and one adult-focused row; four rows without reported protein validation and two with tagged/heterologous protein evidence. The latter does not establish endogenous receptor-protein loss. (**C**) The studies provide methodological examples of allele characterization, phenotyping and complementary validation. The relevance labels are author-assigned qualitative categories, not a validated evidence-strength ranking. None directly tests zebrafish *oprm1* or establishes protection from opioid addiction or respiratory toxicity. ADGRA2, adhesion G-protein-coupled receptor A2; ADGRG1, adhesion G-protein-coupled receptor G1; CRISPR, clustered regularly interspaced short palindromic repeats; GPCR, G-protein-coupled receptor.

Targeted disruption of *oprm1* could help determine the receptor dependence of these phenotypes. The GPCR genome-editing studies in Figure 5 provide methodological precedent for this work, including allele confirmation, transcript analysis, phenotyping, and orthogonal validation. None directly targets an opioid receptor, so their inclusion establishes the feasibility of developing and testing GPCR loss-of-function models rather than evidence for MOR-mediated addiction or overdose. The distinction is also important for the cited CRISPR study of MOR: Shimizu and Shiraki used genome editing to tag endogenous receptors in human SH-SY5Y cells [93]. Direct application to zebrafish *oprm1* would require independent validation of the edited allele, receptor function, and relevant behavioral or respiratory-related readouts.

Combining validated genetic models with neural imaging and endpoint-specific pharmacology could help connect opioid exposure to changes in arousal and oxygen homeostasis. Zebrafish can support mechanistic screening at a scale that complements mammalian studies, but a protective effect on a larval endpoint requires confirmation in an appropriate mammalian respiratory assay before it is interpreted as protection from opioid-induced ventilatory depression.

## 6. Shared vs. Divergent Pathways/Molecular and Cellular Pathways

### 6.1. Shared vs. Divergent Pathways

**Figure 6:**
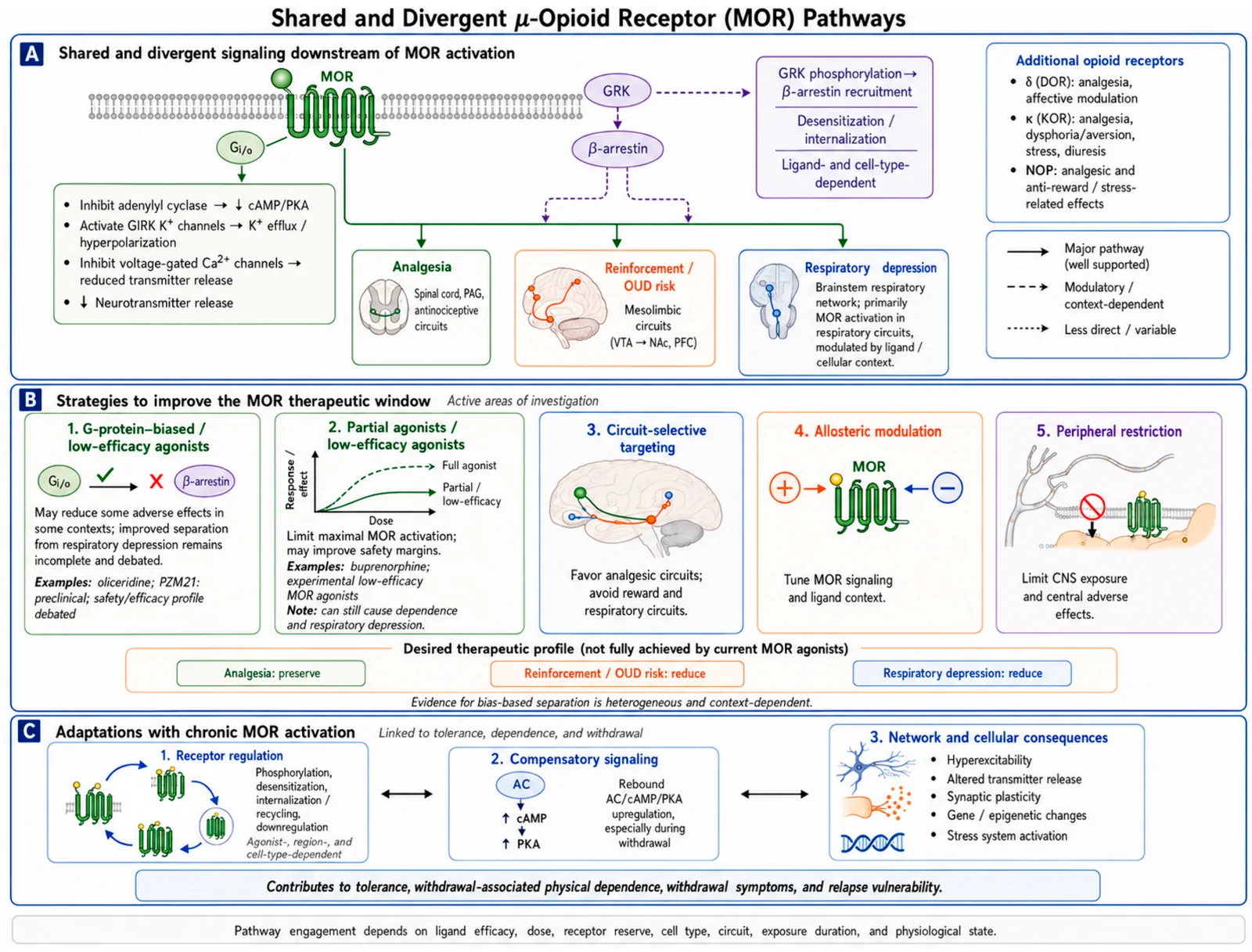
Shared and divergent μ-opioid receptor pathways underlying analgesia, reinforcement, respiratory depression, and chronic adaptations. μ-opioid receptor (MOR) activation engages shared intracellular signaling mechanisms, including G_i/o_ signaling, inhibition of adenylyl cyclase/cAMP/PKA signaling, activation of GIRK K^+^ channels, inhibition of voltage-gated Ca^2+^ channels, and reduced neurotransmitter release. These shared pathways produce divergent physiological outcomes depending on ligand efficacy, dose, receptor reserve, cell type, circuit localization, exposure duration, and physiological state. MOR activation in spinal and supraspinal antinociceptive circuits contributes to analgesia, whereas engagement of mesolimbic circuitry contributes to reinforcement, incentive learning, and opioid use disorder risk. MOR activation in brainstem respiratory networks contributes to respiratory depression, with β-arrestin recruitment and receptor regulation shown as ligand- and cell-type-dependent modulatory processes rather than universal drivers. Strategies under investigation to improve the MOR therapeutic window include G-protein-biased or low-efficacy agonists, partial agonists, circuit-selective targeting, allosteric modulation, and peripheral restriction. These approaches aim to preserve analgesia while reducing reinforcement/OUD risk and respiratory depression, but this desired therapeutic profile has not been fully achieved by current MOR agonists. With chronic MOR activation, agonist-, region-, and cell-type-dependent receptor regulation, compensatory/rebound AC/cAMP/PKA signaling, and network-level plasticity contribute to tolerance, withdrawal-associated physical dependence, withdrawal symptoms, and relapse vulnerability. This framework summarizes the overlap and divergence among molecular, cellular, and circuit mechanisms that shape therapeutic and adverse opioid effects.

Opioid effects (analgesia, reward/addiction, and respiratory depression) are mediated by overlapping, yet distinct molecular and cellular pathways. Shared pathways predominantly involve μ-opioid receptor (MOR) activation, G_i/o_ protein signaling, suppression of adenylyl cyclase, modulation of ion channels, and β-arrestin recruitment. These mechanisms contribute to analgesia, reward, tolerance, and respiratory depression, highlighting why opioids simultaneously produce therapeutic and adverse effects [14,36]. At the cellular level, MOR activation inhibits neuronal excitability and neurotransmitter release. Its circuit-level effects depend on the targeted cells: inhibition of excitatory respiratory neurons can suppress breathing, whereas inhibition of GABAergic inputs can disinhibit reward and antinociceptive circuits [15,28,79]. Divergent pathways emerge from circuit-specific localization and signaling bias (Figure 6A). MOR signaling in nociceptive circuits contributes to analgesia, while engagement of respiratory-control circuits contributes to ventilatory suppression. These circuit differences cannot be reduced to G-protein versus β-arrestin signaling; a bias measurement alone does not establish respiratory safety [19,33]. Similarly, reward-related effects involve mesolimbic dopamine modulation, which is largely distinct from the brainstem circuits controlling breathing, though partially overlapping in stress and limbic integration. By mapping shared and divergent pathways, researchers can identify therapeutic targets that maximize analgesia while minimizing addiction and overdose risk. This framework informs the development of G-protein-biased opioids, allosteric modulators, and circuit-specific interventions (Figure 6B).

### 6.2. Potential for Dissociating Analgesia from Addiction and Respiratory Depression

Efforts to separate analgesia from reinforcement and respiratory depression must account for the circuit and exposure conditions under which each effect is measured. The receptor-regulatory differences described in Section 2.5 motivate biased-agonist strategies, but improved safety cannot be inferred from signaling bias alone [10,19,33]. Intrinsic efficacy, receptor reserve, pharmacokinetics, and tissue distribution also need to be considered when comparing therapeutic and adverse effects.

### 6.3. Chronic Opioid Exposure

Chronic opioid use induces widespread cellular and molecular adaptations that impact analgesia, reward, and overdose susceptibility. Prolonged MOR activation can produce receptor desensitization and changes in trafficking or expression that contribute to tolerance; these adaptations depend on the agonist, cell type, and circuit [14,35]. These adaptations are often accompanied by upregulation of adenylyl cyclase and protein kinase A signaling, altering neuronal excitability and synaptic transmission across the spinal cord, mesolimbic dopamine system, and brainstem respiratory centers [56]. Chronic exposure also induces structural plasticity, including changes in dendritic spine density in the nucleus accumbens, prefrontal cortex, and amygdala, which enhance reward sensitivity and compulsive drug-seeking behaviors [58] (Figure 6C). In respiratory circuits, prolonged opioid exposure may lead to partial tolerance to respiratory depression, yet high-potency opioids like fentanyl can still override compensatory mechanisms, increasing overdose risk. Additionally, neuroinflammatory responses and glial activation contribute to maladaptive plasticity, altering pain processing, reward sensitivity, and autonomic regulation [100]. Understanding these chronic adaptations is critical for designing strategies that maintain analgesia while minimizing dependence and overdose potential, such as biased agonists, peripherally restricted opioids, or interventions targeting specific plasticity mechanisms.

### 6.4. Neuroinflammation

Neuroinflammation is increasingly recognized as a key modulator of opioid effects, influencing analgesia, reward, tolerance, and overdose susceptibility. Chronic opioid exposure activates microglia and astrocytes, leading to the release of pro-inflammatory cytokines such as IL-1β, TNF-α, and IL-6, which alter neuronal excitability and synaptic transmission across pain, reward, and autonomic circuits [100,101]. In the spinal cord, glial activation reduces opioid analgesic efficacy, contributing to tolerance, while in mesolimbic pathways, neuroimmune signaling can alter reward processing and drug-seeking behavior [100,102]. In brainstem respiratory centers, neuroinflammatory changes may impair compensatory ventilatory responses, exacerbating opioid-induced respiratory depression and increasing overdose risk [100]. Targeting neuroinflammation offers a promising approach to dissociate therapeutic and adverse opioid effects. Pharmacological agents that inhibit glial activation, modulate cytokine signaling, or normalize neuroimmune interactions can enhance analgesia, reduce tolerance, and potentially protect against respiratory depression and addictive behaviors [101]. Zebrafish and rodent models have been instrumental in linking opioid-induced neuroinflammation with behavioral and physiological outcomes, providing a translational platform for preclinical testing of anti-inflammatory interventions.

### 6.5. Genetic and Epigenetic Regulation

Genetic and epigenetic mechanisms play a central role in shaping opioid responses, influencing analgesia, reward, tolerance, and overdose risk. Variants in genes encoding the μ-opioid receptor (*oprm1*), dopamine receptors, serotonin transporters, and cytochrome P450 enzymes can modulate receptor sensitivity, signaling bias, and metabolism, thereby altering susceptibility to addiction and respiratory depression [90,91]. Chronic opioid exposure triggers epigenetic remodeling, including DNA methylation, histone modifications, and microRNA regulation, which modify transcriptional programs in pain, reward, and autonomic circuits [58,59]. For example, ΔFosB accumulation and changes in CREB activity contribute to transcriptional plasticity, with effects on reward that depend on the brain region and experimental context [58,59,61]. MicroRNAs can regulate MOR expression and contribute to adaptations associated with chronic opioid exposure [62]; their contribution to differential vulnerability to respiratory depression requires direct testing. Understanding these mechanisms provides insight into individual variability in opioid effects and highlights potential targets for precision therapeutics, including epigenetic modulators and gene-targeted interventions that could dissociate analgesia from addictive and lethal side effects.

## 7. Therapeutic Targets, Interventions, and Research Gaps

### 7.1. Current Pharmacotherapies

Current pharmacotherapies for opioid addiction and overdose primarily include opioid agonist, partial agonist, and antagonist strategies. Methadone, a long-acting full μ-opioid receptor (MOR) agonist, reduces withdrawal symptoms and cravings while providing stable plasma levels that minimize euphoric peaks [103]. Buprenorphine, a partial MOR agonist with high receptor affinity, effectively mitigates withdrawal and craving while carrying a lower risk of respiratory depression compared to full agonists [104]. Naloxone, a rapid-acting MOR antagonist, is the standard treatment for acute opioid overdose, reversing life-threatening respiratory depression within minutes [105]. Because opioid effects can outlast naloxone, repeated dosing may be needed when respiratory depression persists or recurs. Naltrexone, a long-acting oral or injectable antagonist, is used for relapse prevention, but requires prior detoxification due to the risk of precipitated withdrawal [103]. Despite these therapies, limitations persist, including incomplete prevention of overdose, variable adherence, and abuse liability of agonist therapies. Current pharmacotherapies primarily target MOR, leaving reward, tolerance, and neuroinflammatory pathways largely unaddressed. Consequently, there is growing interest in adjunctive treatments and novel interventions that can dissociate analgesia from addiction and respiratory depression, highlighting the need for next-generation therapies.

### 7.2. Emerging Therapies

Emerging therapies aim to expand treatment options for opioid addiction and overdose by targeting alternative mechanisms beyond classical MOR pharmacology. Opioid vaccines aim to elicit antibodies that bind the target opioid in the bloodstream and reduce its central nervous system penetration. Fentanyl conjugate vaccines attenuated fentanyl-induced hotplate antinociception in mice and rats and reduced brain fentanyl concentrations and respiratory depression, assessed by oxygen saturation, in rats [106]. In male rhesus monkeys, a fentanyl-targeted vaccine selectively reduced fentanyl potency in antinociception and schedule-controlled responding assays [107]. In a small rhesus-monkey study, a fentanyl-targeted vaccine reduced fentanyl-versus-food choice, although the magnitude and persistence of the effect varied between animals [108]. Adjunctive therapies targeting neuroinflammation, glial activation, or epigenetic mechanisms are also under investigation, aiming to reduce tolerance, dependence, and long-term maladaptive plasticity associated with chronic opioid use [101]. Additionally, combination pharmacotherapies, such as opioid agonists with modulators of dopaminergic or glutamatergic signaling, are being explored to dissociate analgesia from reward, potentially reducing addiction liability while maintaining pain control. These emerging approaches highlight the potential for mechanism-driven, personalized interventions that address both analgesic efficacy and overdose prevention.

### 7.3. Gene Editing: CRISPR vs. RNA-Based Therapy

Gene-editing technologies offer a novel approach to modulate opioid receptor function and addiction susceptibility. CRISPR-Cas9 provides tools to modify genes encoding opioid receptors. For example, endogenous MOR was tagged in human SH-SY5Y cells to examine receptor regulation during sustained opioid exposure [93]. Extending receptor-specific editing to validated in vivo models could help test contributions to analgesia, reward, and respiratory-related phenotypes. By selectively disrupting MOR in mesolimbic or brainstem circuits, CRISPR approaches could theoretically attenuate addiction or overdose risk while preserving peripheral analgesia. As an example of complementary target validation, pharmacological inhibition and isoform-targeted CRISPR perturbation identified spinal Hsp90 isoforms that regulate opioid antinociception in mice [109]. This work perturbed a non-receptor regulator and does not establish an *oprm1*-knockout phenotype. RNA-based therapies, including antisense oligonucleotides (ASOs) and small interfering RNAs (siRNAs), offer reversible suppression of MOR expression or downstream signaling molecules. These strategies modulate RNA without intentionally making permanent genomic changes, although their duration, specificity, and off-target effects still require evaluation [110]. Both approaches face translational challenges, including delivery across the blood–brain barrier, cell-type specificity, and immune responses. Zebrafish and rodent models provide essential platforms to test efficacy, safety, and circuit-specific outcomes before clinical application. While still experimental, gene-editing and RNA-based interventions hold promise for precision therapies that target molecular determinants of opioid addiction and overdose, complementing conventional pharmacotherapy and offering potential long-term solutions to the opioid crisis.

### 7.4. Allosteric Modulators

Allosteric modulators represent a promising strategy to fine-tune μ-opioid receptor (MOR) activity without directly competing with endogenous ligands. These compounds bind to distinct receptor sites, modulating the receptor’s response to opioids, either enhancing (positive allosteric modulators, PAMs) or reducing (negative allosteric modulators, NAMs) signaling. MOR-positive allosteric modulators can enhance responses to orthosteric agonists, providing a rationale for testing whether analgesia can be achieved at lower opioid doses without increasing adverse effects [111,112]. Conversely, negative allosteric modulators may dampen receptor overactivation, mitigating reward-driven behaviors and decreasing overdose risk. Selected allosteric modulators show receptor-subtype selectivity, but circuit selectivity and reduced adverse effects require direct demonstration [111,113]. In mouse studies, selected MOR-positive allosteric modulators enhanced opioid antinociception without potentiating the measured somatic withdrawal, analgesic tolerance, or respiratory effects, although the response depended on the modulator, opioid, and assay [112]. Allosteric modulators alter receptor conformations and orthosteric-ligand responses; their effects on signaling bias and the separation of therapeutic and adverse effects depend on the compound and experimental context. A related but distinct strategy uses bitopic ligands that engage both the orthosteric pocket and the conserved sodium-binding region of MOR; fentanyl-derived compounds designed in this way altered receptor signaling and were evaluated in preclinical mouse assays [114].

### 7.5. Biased Agonists Aim to Reduce Respiratory Depression

Biased MOR agonists have been investigated as a means to improve the separation between analgesic and adverse effects. Preclinical studies of TRV130 and PZM21 supported further evaluation of this approach [10,17], but the response depends on the ligand, dose, assay, and physiological context. Reduced intrinsic efficacy is a separate property that must be considered alongside measured signaling bias. Neither property establishes a favorable therapeutic window without direct comparison of analgesia, respiratory effects, or reinforcement at relevant exposures. The conflicting β-arrestin knockout findings discussed in Section 5.5 further limit a simple mechanistic explanation for improved safety [18,19]. The extent to which signaling bias improves clinical safety remains under investigation; any benefit should be defined by measured outcomes rather than assumed from pathway preference.

## 8. Limitations, Future Direction, Challenges

Despite significant advances in understanding opioid neurobiology and developing interventions to reduce addiction and overdose, several limitations and challenges remain in both preclinical and clinical research. One key limitation is the translational gap between animal models and human physiology. Zebrafish provide mechanistic insights into opioid receptor signaling and behavior, but species-specific physiology and behavioral repertoires can complicate extrapolation to humans [2,5]. Zebrafish larvae, for example, rely on cutaneous respiration, which differs fundamentally from mammalian pulmonary control, making dose–response relationships and respiratory depression thresholds challenging to directly translate. Similarly, complex cognitive and social factors influencing addiction in humans are difficult to model in animals. Another challenge lies in the complexity of opioid receptor signaling and neurocircuitry. Shared pathways underlie both therapeutic and adverse effects, and crosstalk with dopaminergic, glutamatergic, serotonergic, noradrenergic, and endocannabinoid systems introduces further variability. Although biased agonists, allosteric modulators, and gene-editing strategies offer mechanistic approaches to dissociate analgesia from reward or respiratory depression, off-target effects, receptor redundancy, and compensatory adaptations remain significant hurdles [10,33]. Chronic opioid exposure induces epigenetic and neuroinflammatory changes that are only partially reversible, complicating long-term intervention strategies. Pharmacological interventions also face clinical and societal barriers. Current therapies, including methadone, buprenorphine, and naloxone, face implementation barriers such as stigma and limited access; adherence and diversion are additional concerns for agonist maintenance therapies. Novel strategies such as immunotherapies, RNA-based therapies, and CRISPR approaches are still largely experimental and raise ethical, safety, and regulatory concerns, particularly regarding irreversible genetic modifications or off-target immunogenic effects. Moreover, individual variability in genetics, epigenetics, and co-morbidities adds complexity to precision medicine approaches. Looking forward, future research must focus on integrated, multi-level strategies. Combining preclinical mechanistic studies with human neuroimaging, genetics, and pharmacokinetic modeling can help bridge translational gaps. Advances in circuit-specific targeting, G-protein-biased agonists, and allosteric modulators offer promising avenues to maximize analgesia while minimizing addiction and overdose. Zebrafish and rodent models can be further leveraged for high-throughput screening, real-time neural imaging, and gene-editing validation, providing mechanistic insights into dose–response relationships, neuroinflammation, and plasticity. Beyond conventional locomotor readouts, unsupervised profiling of zebrafish social behavior has distinguished drug-associated phenotypes, including effects of dopamine-receptor agonists, providing a methodological precedent for high-content behavioral screening [115]. Additionally, interdisciplinary approaches integrating pharmacology, genomics, behavioral neuroscience, and computational modeling are critical to predict individual risk, optimize dosing, and identify novel therapeutic targets. Addressing societal challenges, including healthcare access, education, and stigma reduction, is essential for translating scientific advances into public health impact. In summary, while progress in understanding opioid neurobiology and developing targeted therapies is substantial, limitations in model systems, receptor complexity, chronic adaptations, and clinical implementation highlight the need for innovative, mechanism-driven, and multi-level strategies. Overcoming these challenges will be essential to effectively reduce opioid addiction and overdose while preserving analgesic efficacy.

## 9. Conclusions

Opioid addiction and overdose remain a major public health crisis, driven by the convergence of potent μ-opioid receptor (MOR) pharmacology, mesolimbic reward circuitry, and complex neuromodulatory networks. Understanding the neurobiological pathways underlying opioid effects—from analgesia to reward, tolerance, and respiratory depression—has illuminated both shared and divergent mechanisms that govern therapeutic and adverse outcomes. MOR signaling engages G-protein-dependent effectors and β-arrestin-dependent receptor regulation. Their physiological consequences depend on ligand efficacy, exposure, and circuit context; respiratory suppression cannot be assigned universally to β-arrestin recruitment. Parallel engagement of dopaminergic, glutamatergic, serotonergic, noradrenergic, and endocannabinoid systems further modulates reward, mood, and autonomic functions, complicating the separation of analgesic and deleterious effects. Preclinical models, including rodents and zebrafish, have been instrumental in dissecting these pathways. Zebrafish provide high-throughput, genetically tractable, and optically accessible platforms to study opioid-induced reward, locomotor activity, and hypoxia. Conditioned place preference, locomotor assays, and respiratory-related measurements provide distinct readouts of opioid effects. Combining these assays with validated receptor-specific genetic models could strengthen causal interpretation, while mammalian studies remain necessary to establish relevance to ventilatory depression. Current therapies, including methadone, buprenorphine, and naloxone, effectively reduce withdrawal, craving, and acute respiratory depression, but limitations persist, such as adherence challenges, risk of diversion, and incomplete mitigation of overdose risk. Emerging approaches, including G-protein-biased agonists, allosteric modulators, gene-editing strategies, RNA-based interventions, and immunotherapies, offer mechanistic avenues to dissociate analgesia from reward and respiratory depression, highlighting the promise of precision therapeutics. Despite these advances, challenges remain. Translational gaps between animal models and humans, circuit complexity, chronic neuroadaptations, and societal barriers, including access, stigma, and individual variability, complicate implementation. Future research integrating mechanistic neuroscience, pharmacology, genetics, epigenetics, and computational modeling, along with interdisciplinary collaboration, will be essential to identify novel therapeutic targets, optimize dosing, and improve patient outcomes. In conclusion, a comprehensive understanding of opioid neurobiology, combined with innovative therapeutic strategies and translational research, holds the key to mitigating the opioid crisis. By targeting shared and divergent pathways at molecular, cellular, and circuit levels, it is increasingly possible to maximize analgesia while minimizing addiction and overdose, offering hope for safer pain management and improved public health.

## Data Availability

No new datasets were generated in this review. All information is included in the manuscript and its cited references. Further inquiries may be directed to the corresponding author.
